# THE EFFECT OF EARLY SCHOOLING EXPERIENCES AND ADHD POLYGENIC RISK ON COGNITIVE PERFORMANCE IN LATE LIFE

**DOI:** 10.1093/geroni/igad104.2239

**Published:** 2023-12-21

**Authors:** Marrium Mansoor, Benjamin Katz

**Affiliations:** Virginia Tech, Blacksburg, Virginia, United States; Virginia Tech, Blacksburg, Virginia, United States

## Abstract

Attention-Deficit/Hyperactivity Disorder (ADHD) is a neurodevelopmental disorder characterized by executive function (EF) deficits that may impact academic performance, workplace achievement, and other outcomes throughout adulthood. Although 6.76% of adults worldwide may be affected by ADHD, the interplay of early life factors and genetic risk is still not fully understood, especially in the context of cognitive function in later life. While literature suggests that early life experiences may moderate the links between genetic risk for ADHD and later life cognition, few studies have examined these links quantitatively. This research included ~7000 participants from the Health and Retirement Study, a nationally-representative longitudinal study of 37,000 Americans. A moderation analysis was conducted to examine the effect of repeating a year of school before the age of 18 on the relationship between ADHD risk and EF performance as measured by scores on Number Series and Serial 7s tests. Results showed that ADHD genetic risk significantly predicted performance on EF measures; furthermore, the interaction term was significant while controlling for demographics, self-rated health, and maternal education. However, only older adults with low polygenic risk for ADHD displayed differential cognitive performance as a function of early-life academic adversity, while those with high polygenic risk displayed reduced performance on EF-related measures regardless of whether they repeated a grade. This finding suggests that the effect of genetic risk for ADHD on later life EF performance may remain stable across earlier educational experiences and informs future work examining the impact of gene-environment interactions on cognition in later life.

